# Effect of Marine Bacteria and Ulvan on the Activity of Antioxidant Defense Enzymes and the Bio-Protection of Papaya Fruit against *Colletotrichum gloeosporioides*

**DOI:** 10.3390/antiox8120580

**Published:** 2019-11-23

**Authors:** Roberto G. Chiquito-Contreras, Bernardo Murillo-Amador, Saul Carmona-Hernandez, Cesar J. Chiquito-Contreras, Luis G. Hernandez-Montiel

**Affiliations:** 1 Facultad de Ciencias Agrícolas, Universidad Veracruzana, Campus Xalapa, Circuito Universitario Gonzalo Aguirre Beltrán s/n, Zona Universitaria, Xalapa 91090, Ver., Mexico; contrerasch@yahoo.com (R.G.C.-C.); ingesaul93@gmail.com (S.C.-H.); cchiquito@uv.mx (C.J.C.-C.); 2 Centro de Investigaciones Biológicas del Noroeste (CIBNOR), Calle Instituto Politécnico Nacional 195, Col. Playa Palo de Santa Rita Sur, La Paz 23096, B.C.S., Mexico; bmurillo04@cibnor.mx

**Keywords:** *Stenotrophomonas rhizophila*, *Bacillus amyloliquefaciens*, biocontrol, anthracnose

## Abstract

Anthracnose, caused by *Colletotrichum gloeosporioides*, is one of the most important diseases in papaya fruit. Its control has been achieved with synthetic fungicides, but the application of marine bacteria and the sulphated polysaccharide ulvan (structural description: β[1,4]-D-GlcA-α[1,4]-L-Rha 3 sulfate, β[1,4]-L-IdoA-α[1,4]-L-Rha 3 sulfate, β[1,4]-D-Xyl-α[1,4]-L-Rha 3 sulfate, and β[1,4]-D-Xyl 2-sulfate-α[1,4]-L-Rha 3 sulfate) from *Ulva* sp. can be an alternative in the use of agrochemicals. Thus, the objective of this study was to assess the effect in vitro and in vivo of two marine bacteria, *Stenotrophomonas rhizophila* and *Bacillus amyloliquefaciens,* and ulvan in papaya fruit’s bio-protection against *C. gloeosporioides*. The capacity of marine bacteria to inhibit mycelial growth and phytopathogen spore germination in vitro through volatile organic compounds (VOCs) and carbohydrate competition was evaluated. Fruit was inoculated with bacteria, ulvan, and *C. gloeosporioides* and incubated at 25 °C and 90% of relative humidity (RH) for seven days. Disease incidence (%), lesion diameter (mm), and antioxidant defense enzyme activity (such as superoxide dismutase (SOD), catalase (CAT), and peroxidase (POD) were quantified. In vitro, *C. gloeosporioides* was inhibited by *S. rhizophila* and *B. amyloliquefaciens*. In vivo, disease incidence and the lesion diameter of anthracnose on papaya fruit were significantly reduced by marine bacteria and ulvan. Antioxidant defense enzyme activity played an important role in fruit bio-protection against *C. gloeosporioides*. The application of marine bacteria and ulvan can be an alternative in the sustainable postharvest management of papaya.

## 1. Introduction

Worldwide, the loss of fruit at postharvest caused by phytopathogens is estimated to reach 50% of total production [1]. Papaya is a fruit with high demand in the world market; however, due to its water content, it is susceptible to fungal diseases, of which anthracnose is one of the main diseases causing economic losses [2]. The fungus *Colletotrichum gloeosporioides* is the causal agent of anthracnose, occupying eighth place in the classification of important plant phytopathogens [3,4].

Synthetic fungicides constitute the main method of controlling anthracnose; nevertheless, the use of these agrochemicals generates resistance to phytopathogens, besides the adverse effects on the environment and human and animal health [5]. The presence of synthetic fungicide residuals on food has led to the search for alternatives to control phytopathogens, where the use of microorganisms as biocontrol agents and the application of algae as resistance inductors in plants is an important option to reduce or replace agrochemicals [6,7].

The use of bacteria as biocontrol agents has been increasing because of their capacity to decrease diseases caused in fruit by fungi [8]. Antagonist bacteria are generally isolated from plants, fruit, or soil [9], but the isolation of antagonistic microorganisms in extreme environments has been studied little. Bibi et al. [10,11] mentioned that bacteria isolated from marine environments play an important role in plant protection against phytopathogens because of their capacity to produce diverse antimicrobial substances and induce host resistance. Some reports have indicated the efficiency of bacteria, such as *Stenotrophomonas rhizophila* and *Bacillus amyloliquefaciens*, in the biocontrol of phytopathogen fungi in peach [12], mango [13], and apple [14] postharvest. Within the main antagonistic mechanisms of bacteria against phytopathogens is the competition for carbon sources, volatile organic compounds (VOCs), induction of host defense antioxidants, lytic enzyme production, and siderophores, among others [15]. On the other hand, in the last years, the intensity of the search for new marine bio-active compounds has increased [16], mainly those coming from diverse marine algae, which have had different applications within the food, cosmetology, and pharmaceutical industries [17,18] because of their polysaccharide activities as anticoagulants, antiviral, antitumoral, and immunomodulators, among others [19]. Algal polysaccharides are probably the most abundant molecules in the ocean, with great molecular biodiversity and different biological activities that have been little studied for their application in agriculture [20], as antifungal agents against disease or spoilage organisms of economic importance [16].

*Ulva* sp. is a green marine alga that has been studied for its polysaccharide activities, such as antiviral [21], antibacterial [22], antioxidant [23], antiparasitic, larvicidal [24,25], anti-inflammatory [26], and anti-coagulant activity [27], among others. In agriculture, different species of *Ulvan* have been used as growth promoters of different plants [28,29] and their antifungal effects have been generally assessed in vitro [30]. Several studies are available on the protector effects of polysaccharide ulvan in vivo on plants or fruits; for example, it reduced the leaf spot caused by *C. gloeosporioides* in apples [31], decreased rotting in melon fruit caused by *Fusarium proliferatum* [32], and protected *Medicago trucantula* against *C. trifolii* [33]. Ulvan polysaccharide protects fruit because it induces the acquired resistance system (ARS), increasing compounds in the host, such as catalase, peroxidase, oxidase polyphenol activity, phenolic compounds, and pathogenesis-related (PR) proteins [34,35].

Despite the efficient bio-protection performed individually by marine bacteria and ulvan against different phytopathogens in different hosts and as alternative treatments to the use of synthetic fungicides, to our knowledge, no study has been undertaken yet that deals with their effect on papaya fruit conservation. Therefore, the objective of this study was to assess the effect of marine bacteria and ulvan in postharvest on the antioxidant defense enzyme activity in the host and the bio-protection capacity of papaya against anthracnose caused by *C. gloeosporioides*.

## 2. Materials and Methods

### 2.1. Colletotrichum Gloeosporioides

The phytopathogen fungus was isolated previously from papaya fruit with anthracnose [36] and provided by the Phytopathology Laboratory at Centro de Investigaciones Biológicas del Noroeste (CIBNOR). The fungus was activated in potato dextrose agar (PDA, Merck, Germany) at 25 °C for seven days. The *C. gloeosporioides* concentration was adjusted to 1 × 10^4^ spores/mL using a haemocytometer.

### 2.2. Marine Bacteria

Two marine bacteria, *Bacillus amyloliquefaciens* (strain 1R1CB) and *Stenotrophomonas rhizophila* (strain KM02) [13], were provided by CIBNOR Phytopathology Laboratory. Bacteria were cultured in trypticase soy broth (TSB, Difco, BD, Franklin Lakes, NJ, USA) at 25 °C and 125 rpm for 24 h. The concentration of each bacterium was adjusted to 1 × 10^6^ cell/mL using a digital spectrophotometer (Thermo Spectronic Genesys 20, Thermo Fisher Scientific, Inc., Waltham, MA, USA) calibrated to a 660-nm wavelength and an absorbance of 1.0.

### 2.3. Polysaccharide Ulvan

The ulvan (#ULV010, obtained from *U. armoricana* polysaccharide hydrolysis, OligoTech^®^, Elicityl Ltd., Crolles, France) solution was prepared at 2 mg/mL using sterile deionized water [32].

### 2.4. Effect of Ulvan on Spore Germination of C. gloeosporioides

Erlenmeyer flasks containing 50 mL of potato dextrose broth (PDB, Difco, BD, Franklin Lakes, NJ, USA) were inoculated with 500 µL of a suspension adjusted to 1 × 10^4^ spores/mL of *C. gloeosporioides* and 2 µL of ulvan. Flasks were incubated at 25 °C and 125 rpm. Aliquots of 10 µL were collected from each flask at 6, 12, 18, and 24 h to determine the spore germination rate of *C. gloeosporioides* with an optical microscope (CARL ZEISS, Primo Star, Oberkochen, Germany). In total, 100 spores were observed per treatment, and a spore was considered germinated when the germ tube was equal to or greater than the spore size. Ten replicates were performed per treatment, and the experiment was repeated twice.

### 2.5. In Vitro Antagonistic Activity of Marine Bacteria to C. gloeosporioides

#### 2.5.1. Antifungal Activity

Plugs from a 7-day culture of *C. gloeosporioides* in PDA were deposited in the center of a Petri dish with PDA; subsequently, marine bacteria cells were placed beside the mycelial plug at a distance of 2 cm. A group of Petri dishes were inoculated with the phytopathogen fungus and a synthetic fungicide benomyl (Benlate) at a concentration of 1000 ppm, and another group was inoculated only with *C. gloeosporioides*. The Petri dishes were incubated at 25 °C for seven days. The mycelial growth of the phytopathogen was calculated in mm, as well as the inhibition percentage with the formula described by Ezziyyani et al. [37]: PI = (R1 − R2)/R1 × 100, where “PI” is the mycelial growth inhibition percentage; “R1” is the average radius value of the colony as a reference; and “R2” is the average radius value of the colony inhibited by the marine bacteria. Ten replicates were used per treatment, and the experiment was repeated twice.

#### 2.5.2. Inhibition of C. gloeosporioides for VOCs

The experimental assay was based on the mouth-to-mouth method proposed by Rouissi et al. [38]. Petri dishes with tryptic soy agar (TSA, Difco, BD, Franklin Lakes, NJ, USA) were inoculated in the center with a fungus plug from a 7-day fungus culture in PDA. At the same time, 30 μL of *B. amyloliquefaciens* and *S. rhizophila* (1 × 10^6^ cells/mL) were individually streaked in other dishes containing the same media. The plate was placed mouth-to-mouth, sealed (parafilm), and incubated at 25 °C for seven days. The growth diameter of *C. gloeosporioides* was quantified (mm), and the radial growth reduction was calculated with the equation, I (%) = DC-DT/DC × 100, where I% = mycelium growth inhibition in percentage, DC = mycelium measured in control treatment, and DT = mycelium diameter in the presence of the marine bacterium treatment. The control treatment was represented by Petri dishes inoculated only with *C. gloeosporioides*. Ten replicates were used per treatment, and the experiment was repeated twice.

#### 2.5.3. Competition for Carbohydrates

Starting from fruit at commercial maturity, a concentrate of papaya var. Maradol was made and diluted 1:10 with distilled water and then sterilized in an autoclave at 120 °C for 15 min; after that, 50 mL of the sterile papaya juice medium (SPJ) were placed in Erlenmeyer flasks and inoculated with 500 µL of a suspension of each marine bacteria (1 × 10^6^ cell/mL) and 500 µL of a suspension of *C. gloeosporioides* (1 × 10^4^ spores/mL). The Erlenmeyer flasks were incubated at 25 °C and 125 rpm for 24 h; 5-mL aliquots were taken to determine total carbohydrates (anthrone method [39]), sucrose (3,5-Dinitrosalicylic acid (DNS) method [40]), glucose (glucose oxidase-phenol and 4 aminophenazone GOD/PAP [41]), and fructose (tryptamine hydrochloride [42]). The percentage of germinated *C. gloeosporioides* spores was calculated in 100 spores. Ten replicates were used per treatment, and the experiment was repeated twice.

### 2.6. Effect of B. amyloliquefaciens, S. rhizophila, and Ulvan on the Control of Papaya Fruit Anthracnose

Papaya var. Maradol fruit at commercial maturity was disinfected with sodium hypochlorite at 2% for three minutes, then washed with sterile distilled water and dried in a laminar air flow bench for two hours. Three lesions (3 mm in depth each) were made in each fruit with a sterile needle. Subsequently, each wound was inoculated with 20 µL of each bacterium (1 × 10^6^ cell/mL) and ulvan. They were left to dry for two hours, and then each wound was inoculated with 20 µL of a fungus spore suspension (1 × 10^4^ spores/mL). A fruit group was inoculated with *C. gloeosporioides*, and treated with the synthetic fungicide benomyl at a concentration of 1000 ppm. Another group was inoculated only with the phytopathogen. The fruit was placed in sterile plastic containers at 25 °C and 90% relative humidity (RH) for seven days. The disease incidence (%) and lesion diameter (mm) were quantified. Ten replicates were used per treatment, and the experiment was repeated twice.

#### 2.6.1. Antioxidant Defense Enzymes

##### Sample Collection

After the papaya was inoculated with marine bacteria, ulvan, and/or *C. gloeosporioides*, 1 g of fruit tissues at 6, 12, and 24 h was collected. Samples were deposited in Eppendorf flasks, adding 2 mL of phosphate buffer (pH 7.8), and the tissues was homogenized with a Polytron tissue homogenizer (Brinkman Instruments) for 60 s. Subsequently, they were centrifuged at 8000 rpm at 5 °C for 15 min. After that, the supernatant was recovered, placed in Eppendorf flasks, and stored at −40 °C for subsequent analysis. Catalase (CAT), superoxide dismutase (SOD), and peroxidase (POD) activities were expressed in units (U) per gram of protein (U/g protein). The protein content was quantified by the method described in [43], using bovine serum albumin (BSA) as a standard. Ten replicates were used per treatment, and the experiment was repeated twice.

##### Catalase (CAT) Activity

Catalase activity was quantified in 96-well microplates; 10 µL of phosphate buffer of 100 nM (pH 7.0) and 20 µL of the supernatant were deposited in each well and incubated for six minutes. After that, 20 µL of H_2_O_2_ (8.82 M) were added and incubated at room temperature for 20 min. A unit of CAT was defined as the decomposition of 1 µmol of H_2_O_2_ per min. The samples were quantified in a spectrophotometer at an absorbance of 240 nm [44].

##### Superoxide Dismutase (SOD) Activity

The SOD activity was quantified at pH 7.8 in Tris-HCl 0.2 M and sodium carbonate 50 Mm (pH 10), adding 20 µL of the supernatant and 20 µL of xanthine/xanthine oxidase. The superoxide generated by the xanthine/xanthine oxidase was detected by controlling the reduction of nitro blue tetrazolium (NAT) and determined in a spectrophotometer at an absorbance of 505 nm. One unit of SOD was defined as the amount of enzyme that causes an inhibition of 50% in the reduction of NAT [45].

##### Peroxidase (POD) Activity

In 96-well plates, 10 µL of the supernatant and 50 µL guaiacol solution (100 mM of sodium phosphate, pH 6.4, and guaiacol 8 mM) were placed and incubated at 30 °C for 5 min. One unit of POD was defined as an increase of one unit of absorbance per minute under the conditions of the assay. Samples were quantified in a spectrophotometer at an absorbance of 460 nm [46].

### 2.7. Statistical Analysis

Data were processed by a one-way analysis of variance (ANOVA) and Tukey’s test with a significance level of 5%, using the software STATISTICA (version 8.0.360.0 StatSoft Inc., Tulsa, USA) for Windows.

## 3. Results

### 3.1. Inhibition of C. gloeosporioides Spore Germination by Ulvan

Ulvan did not affect the germination of the phytopathogen fungus spores in any of the time intervals assessed (6, 12, 18, and 24 h). At the end of the experiment, *C. gloeosporioides* reached 82% of germinated spores, and phytopathogen fungus and ulvan treatment was 83%; thus, no significant differences were found between treatments with and without ulvan + *C. gloeosporioides*.

### 3.2. In Vitro Antifungal Activity of Marine Bacteria to Phytopathogen Fungus

**Figure 1:**
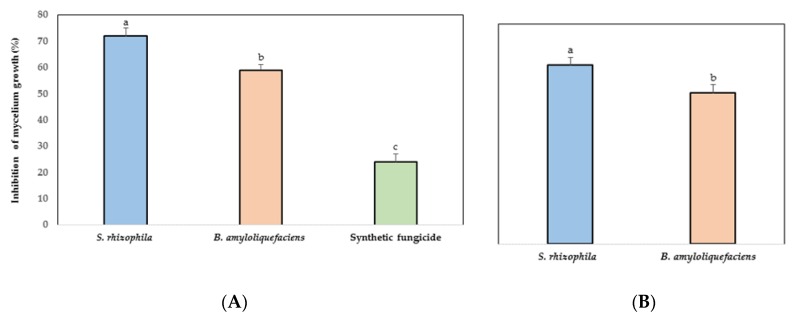
Effect in vitro of marine bacteria on the mycelial growth of *Colletotrichum gloeosporioides*. Inhibition percentage (%) of the phytopathogen fungus by (**A**) direct confrontation; (**B**) volatile organic compounds (VOCs). Data are presented as a mean ± SD (*n* = 10). Columns with different letters were significantly different according to Tukey’s test (*p* < 0.05).

A significant inhibition was observed in the mycelial growth of *C. gloeosporioides* by the effect of *S. rhizophila*, *B. amyloliquefaciens*, and the synthetic fungicide (Figure 1A). *C. gloeosporioides* reduced its growth in 72% by the antagonism of *S. rhizophila*. The marine bacteria were more efficient in terms of the mycelial growth of the phytopathogen fungus compared with the synthetic fungicide.

### 3.3. Inhibition of C. gloeosporioides by VOCs

*S. rhizophila* and *B. amyloliquefaciens* inhibited the mycelial growth of *C. gloeosporioides* significantly through VOCs (Figure 1B). The greatest growth inhibition (65%) of the phytopathogen fungus was achieved by the marine bacteria *S. rhizophila*.

### 3.4. Carbohydrate Competition between Marine Bacteria and C. gloeosporioides

**Figure 2:**
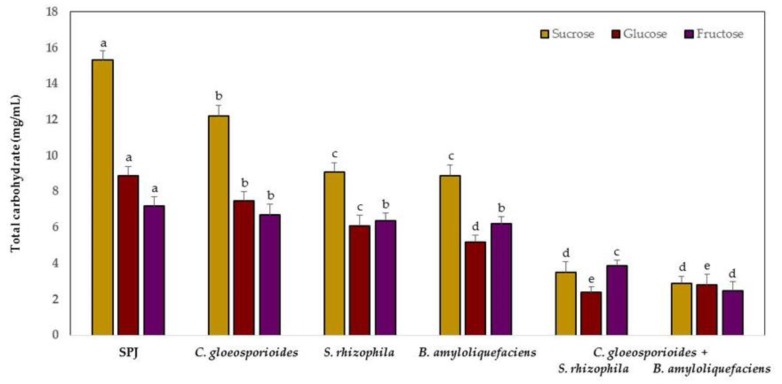
Sucrose, glucose, and fructose contents in sterile papaya juice medium (SPJ) inoculated with marine bacteria and *Colletotrichum gloeosporioides*. Data are presented as mean ± SD (*n* = 10). Columns with different letters were significantly different according to Tukey’s test (*p* < 0.05).

Sucrose, glucose, and fructose decreased rapidly in content in the sterile papaya juice medium (SPJ) when inoculated with the marine bacteria and phytopathogen fungus compared with the medium only with each microorganism (Figure 2). At 24 h, the sucrose content decreased by 81% and fructose by 65% when SPJ was inoculated with *B. amyloliquefaciens* and *C. gloeosporioides*. Glucose reduced by 73% in the presence of *S. rhizophila* and *C. gloeosporioides*.

The spore germination inhibition of *C. gloeosporioides* by *S. rhizophila* and *B. amyloliquefaciens* was quantified at 73% and 65%, respectively. The treatment with only the phytopathogen fungus showed 94% of spore germination at 24 h of inoculation of the SPJ medium.

### 3.5. Bio-Protection of Papaya Fruit Inoculated with Marine Bacteria and Ulvan to Anthracnose

**Figure 3:**
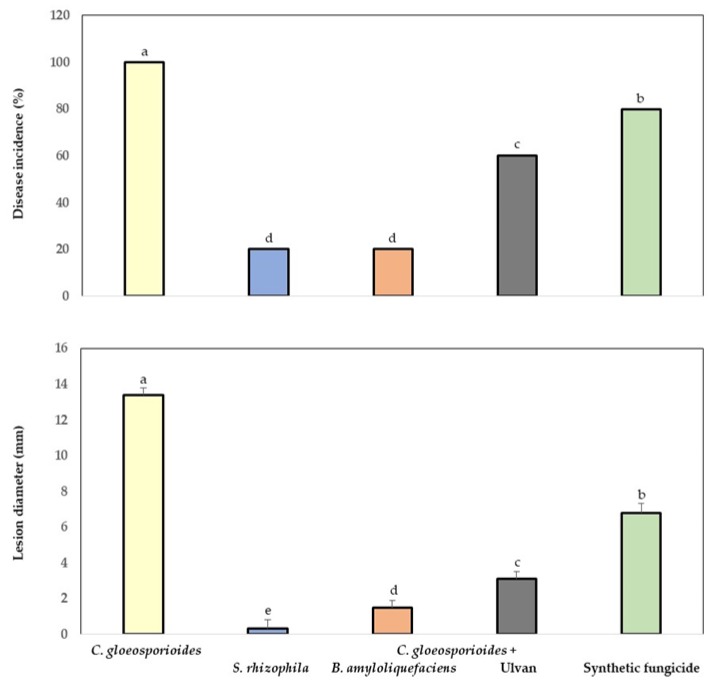
Incidence and lesion diameter caused by *Colletotrichum gloeosporioides* in fruit treated with marine bacteria, ulvan, and synthetic fungicide. Data are presented as a mean ± SD (*n* = 10). The columns with different letters were significantly different according to Tukey’s test (*p* < 0.05).

The fruit inoculated with the phytopathogen fungus, marine bacteria, ulvan, and synthetic fungicide showed significantly lower levels of incidence and lesion diameter (mm) of anthracnose compared with the fruit with only *C. gloeosporioides* (Figure 3). A reduction of 80% was quantified in incidence and 97% in lesion diameter (mm) in fruit inoculated with the phytopathogen fungus and *S. rhizophila*. The application of marine bacteria and ulvan on fruit was more efficient in the control group of anthracnose than in the treatment with synthetic fungicide.

### 3.6. Antioxidant Defense Enzyme Activity in Papaya Fruit Inoculated with Marine Bacteria and Ulvan

**Figure 4:**
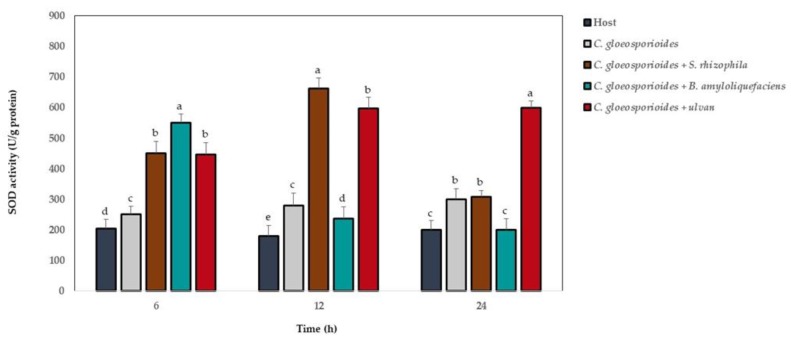
Superoxide dismutase (SOD) activity in papaya fruit inoculated with *Colletotrichum gloeosporioides*, marine bacteria, and ulvan. Data are presented as a mean ± SD (*n* = 10). The columns with different letters were significantly different according to Tukey’s (*p* < 0.05).

The SOD activity showed the highest levels at 6, 12, and 24 h after fruit inoculation with marine bacteria and ulvan, and the phytopathogen fungus showed the highest level in relation to the fruit without microorganisms and ulvan (Figure 4). At 6 h, the highest SOD values were observed in fruit with *C. gloeosporioides* and *B. amyloliquefaciens*; at 12 h, the treatment with the phytopathogen fungus and *S. rhizophila*; and at 24 h, the highest values were observed in the fruit with *C. gloeosporioides* and ulvan.

**Figure 5:**
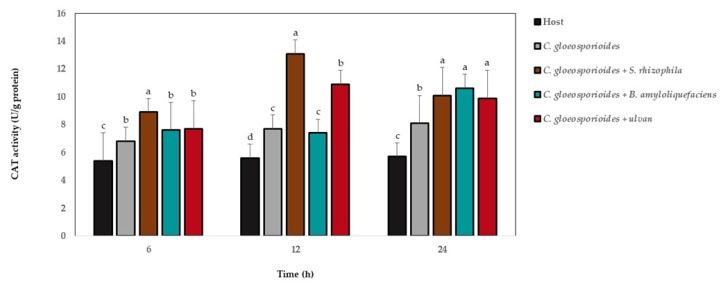
Catalase (CAT) activity in papaya fruit inoculated with *Colletotrichum gloeosporioides*, marine bacteria, and ulvan. Data are presented as a mean ± SD (*n* = 10). Columns with different letters were significantly different according to Tukey’s test (*p* < 0.05).

The CAT activity showed the highest levels in relation to fruit without microorganisms and ulvan at 6, 12, and 24 h after fruit was inoculated with marine bacteria, ulvan, and the phytopathogen fungus (Figure 5). At 6 and 12 h, the highest CAT values were observed in fruit with *C. gloeosporioides* and *S. rhizophila*, and at 24 h, the highest values were observed for treatments with the phytopathogen and each marine bacterium and ulvan.

**Figure 6:**
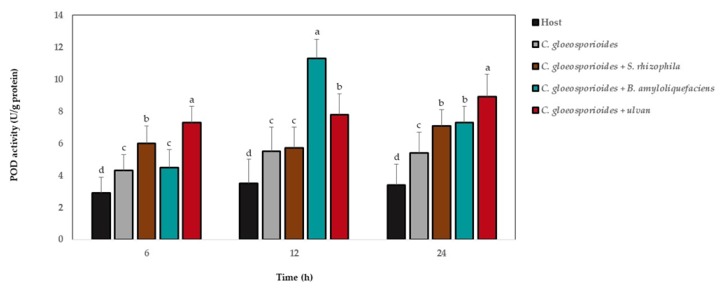
Peroxidase (POD) activity in papaya fruit inoculated with *Colletotrichum gloeosporioides*, marine bacteria, and ulvan. Data are presented as a mean ± SD (*n* = 10). Columns with different letters were significantly different according to Tukey’s test (*p* < 0.05).

The POD activity showed the highest levels in relation to those without microorganism and ulvan at 6, 12, and 24 h after fruit was inoculated with marine bacteria, ulvan, and the phytopathogen fungus (Figure 6). At 6 h, the highest POD values were observed in fruit with *C. gloeosporioides* and ulvan; at 12 h, this was true for the treatment with the phytopathogen fungus and *B. amyloliquefaciens*; and at 24 h, the highest values were observed for fruit with *C. gloeosporioides* and ulvan.

## 4. Discussion

The biological control of postharvest diseases in fruit using antagonistic microorganisms and ulvan as resistance inductors in plants has been an efficient alternative to the use of synthetic fungicides [32,47].

The marine bacteria *S. rhizophila* and *B. amyloliquefaciens* inhibited in vitro mycelial growth of *C. gloeosporioides*. Particularly, the in vitro antagonism of different phytopathogens by *Stenotrophomonas* spp. is related to diverse antagonistic mechanisms that have already been described for some species of this bacterium, of which the production of antimycotic antibiotics, such as maltophilin and xanthobaccins, are macrocyclic lactams that inhibit phytopathogen growth [48,49,50]. A mechanism of *S. rhizophila* is lytic enzyme production, such as proteases, chitinases, and β-1,3-glucanasas, which eliminate fungi when they break the links of mannoproteins, chitin, and glucan contained in their cell wall [51]. *Stenotrophomonas* spp. also inhibit phytopathogens by siderophore synthesis (from Greek *sideros phoros* “iron bearer or carrier”), which are low molecular-weight molecules that act as specific iron chelators, minimizing availability toward other microorganisms that need this essential micronutrient for cellular metabolism, oxygen transport, respiration, and DNA synthesis, among others [52]. The volatile organic compounds produced by *Stenotrophomonas* spp. as β-phenylethanol showed an antimicrobial effect, altering the plasmatic membrane permeability in fungi and sugar and amino acid transport processes [53].

*Bacillus amyloliquefaciens* is a bacterium with a high capacity in vitro to control several phytopathogens [54,55]. The inhibition of *C. gloeosporioides* by this bacillus is related to diverse antagonistic mechanisms, mainly the production of several metabolites, such as 2-propanona and 2-methylpiridine—both VOCs that inhibit fungus growth and stimulate host resistance induction [56,57,58]. Other antimicrobial compounds that this bacillus produces are lytic enzymes, such as β-1,3-glucanases and cellulose, capable of degrading the fungus cell wall [59], antibiotics; polyketides, and cyclic lipopeptides (LPs), mainly from the surfactin, iturin, and fenesin families, which can easily be combined with the lipid layer of the phytopathogen cell membrane, damaging membrane integrity [60,61,62].

In vivo, anthracnose control in papaya fruit by *S. rhizophila*, *B. amyloliquefaciens*, and ulvan was more efficient in comparison to the protection exerted by the synthetic fungicide. The antagonistic microorganisms and ulvan were an alternative to the use and decrease in synthetic applications, which impact the environment and human and animal health negatively, and in the generation of phytopathogen resistance [5,32]. *S. rhizophila* and *B. amyloliquefaciens* share diverse antagonistic mechanisms (lytic enzyme production, VOCs, antibiotics, siderophores, among others) involved in the biocontrol in vivo of diseases in fruit; however, competition for space and nutrients and the induction of host resistance are two important mechanisms that both bacteria have for phytopathogen biocontrol [36]. In this study, the marine bacteria reduced *C. gloeosporioides* spore germination because of sucrose, glucose, and fructose, which were consumed rapidly by *S. rhizophila* and *B. amyloliquefaciens*, decreasing the content of strong carbon sources for their absorption by the phytopathogen. Carbohydrates are of great importance and are useful for spore germination and start the infection process of *C. gloeosporioides* to fruit [63].

On the other hand, bacteria and ulvan have been considered to be inductors of plant resistance [64] and a biotechnological control option of phytopathogens of agricultural importance [33]. In this study, *S. rhizophila*, *B. amyloliquefaciens*, and ulvan protected papaya fruit against anthracnose through the induction of defense antioxidant enzymes, such as SOD, CAT, and POD, whose activities (U/g protein) were higher in fruit treated with marine bacteria or ulvan in relation to the treatment with only the phytopathogen. Thus, the resistance inductors in plants play an important role in disease reduction [35,36]. The function of defense antioxidant enzymes is to protect the host from reactive oxygen species (ROS), such as superoxide anion (O_2_^−^), hydrogen peroxide (H_2_O_2_), and hydroxyl radical (OH^−^), caused by the phytopathogens in their infection process [65,66]. ROS can damage nucleic acids, proteins, carbohydrates, and lipids, and affect cell membrane integrity, directly inactivating key cell functions in plants [67]. For plants, the proportion between the generation and elimination of ROS is transcendental in all cells; thus, defense antioxidant enzymes play an important role in intracellular equilibrium. SOD is a detoxifying enzyme that allows the elimination of ROS in the cell [68]. With respect to POD, it is found in all host cells, protecting them against the destructive activity of H_2_O_2_ through the oxidation of phenolic and enodiolic co-substrates [69,70]. Finally, CAT is found in aerobic cells and protects them from H_2_O_2_, catalyzing its decomposition in O_2_ and H_2_O [71]. Although several studies of bacteria and ulvan exist for defense antioxidant enzyme induction (SOD, CAT, and POD) in plants for their bio-protection against different phytopathogens, such as *Alternaria tenuis* and *Botrytis cinerea* in peach [8], *B. cinerea* in pepper [43], *C. gloeosporioides* in lychee [72], *C. acutatum* in loquat [73], *Penicillum expansum* in apple [35], and *C. trifolli* in *Medicago* sp. [33], this is the first report of the marine bacteria *S. rhizophila* and *B. amyloliquefaciens* and ulvan as resistance inductors in papaya fruit against anthracnose caused by *C. gloeosporioides.*

## 5. Conclusions

*S. rhizophila* and *B. amyloliquefaciens* inhibited the in vitro growth of *C. gloeosporioides*; additionally, the application of both marine bacteria and ulvan on papaya fruit protected them against anthracnose. The mechanism that participated in fruit bio-protection by the antagonistic bacteria and ulvan was competition for sucrose, glucose, and fructose production by VOCs and defense antioxidant enzyme induction, such as SOD, CAT, and POD. Future studies assessing the efficiency of *S. rhizophila*, *B. amyloliquefaciens*, and ulvan at a greater scale should be performed on papaya fruit as an alternative to the use of synthetic fungicides and the sustainable management of agricultural products at postharvest.
